# Supplementary material for: Curcumin Mitigates Gut Dysbiosis and Enhances Gut Barrier Function to Alleviate Metabolic Dysfunction in Obese, Aged Mice
Source: Biology (Basel). 2024 Nov 21;13(12):955. doi: 10.3390/biology13120955 (PMC11726832; doi:10.3390/biology13120955)

biology

MDPI

Supplementary Materials

Table S1. Diet composition for NCD and NCD+CUR

	NCD (DYET# 101845)			NCD+CUR (DYET# 104780)		
**Ingredient**	**kcal/g**	**grams/kg**	**kcal/kg**	**kcal/g**	**grams/kg**	**kcal/kg**
High Nitrogen Casein	3.58	200	716	3.58	200	716
L-Cystine	4	3	12	4	3	12
Sucrose	4	90	360	4	90	360
Cornstarch	3.6	398.562	1434.823	3.6	394.562	1420.4
Dyetrose	3.8	132	501.6	3.8	132	501.6
Soybean Oil	9	70	630	9	70	630
t-Butylhydroqui-none	0	0.014	0	0	0.014	0
Ethoxyquin	0	0.024	0	0	0.024	0
Cellulose	0	50	0	0	50	0
Mineral Mix #210025	0.88	35	30.8	0.88	35	30.8
Vitamin Mix #310025	3.87	10	38.7	3.87	10	38.7
Supplement #410750	3.8	10	38	3.8	10	38
Choline Chloride	0	1.4	0	0	1.4	0
**Curcumin**	0	0	0	0	4	0
Total		1000	**3761.923**		1000	**3747.5**

Table S2. Diet composition for HFHSD and HFHSD+CUR

	**HFHSD (**DYET# 103806)			HFHSD+CUR (DYET# 103864)		
**Ingredient**	**kcal/g**	**grams/kg**	**kcal/kg**	**kcal/g**	**grams/kg**	**kcal/kg**
High Nitrogen Casein	3.58	200	716	3.58	200	716
L-Cystine	4	3	12	4	3	12
Sucrose	4	90	360	4	90	360
Cornstarch	3.6	0	0	3.6	0	0
Dyetrose	3.8	50	190	3.8	50	190
Dextrose	3.64	310.462	1130.1	3.64	306.562	1115.9
Soybean Oil	9	70	630	9	70	630
t-Butylhydroquinone	0	0.014	0	0	0.014	0
Whole Butter (18% Water)	9	181	1629	9	181	1629
Ethoxyquin	0	0.024	0	0	0.024	0
Lard	9	20	180	9	20	180
Cellulose	0	50	0	0	50	0
Mineral Mix #210025	0.88	35	30.8	0.88	35	30.8
Vitamin Mix #310025	3.87	10	38.7	3.87	10	38.7
Supplement #410750	3.8	10	38	3.8	10	38
Choline Chloride	0	1.4	0	0	1.4	0
Dye/Curcumin	0	0.1	0	0	4	0
**Total (excluding water)**			**4954.58**			**4940.386**

Table S3. Sequence of primer used for real-time RT-PCR

S.N.	Primer	Primer sequence (5′-3′)
1.	TNF-α	F-CTGAGGTCAATCTGCCCAAGTAC
		R-CTTCACAGAGCAATGACTCCAAAG
2.	IL-6	F-GAGGATACCACTCCCAACAGACC
		R-AAGTGCATCATCGTTGTTCATACA
3.	IL-1β	F-CAACCAACAAGTGATATTCTCCATG
		R-GATCCACACTCTCCAGCTGCA
4.	IL-10	F-GCT CCT AGA GCT GCG GACTG
		R-TTCCGATAAGGCTTGGCAAC
5.	FGFR4	F-CTTTGGGCAAGTGGTTCGTG
		R-GAGACCAGGTCTGCCAAATC
6.	B-Klotho	F-GATGAAGAATTTCCTAAACCAGGTT
		R-AACCAAACACGCGGATTTC
7.	FXRα	F-TCCGGACATTCAACCATCAC
		R-TCACTGCACATCCCAGATCTC
8.	BSEP	F-TGAATGGACTGTCGGTATCTGTG
		R- CCACTGCTCCCAACGAATG

Figure S1. The original membrane for the western blot is shown in Figure 5.

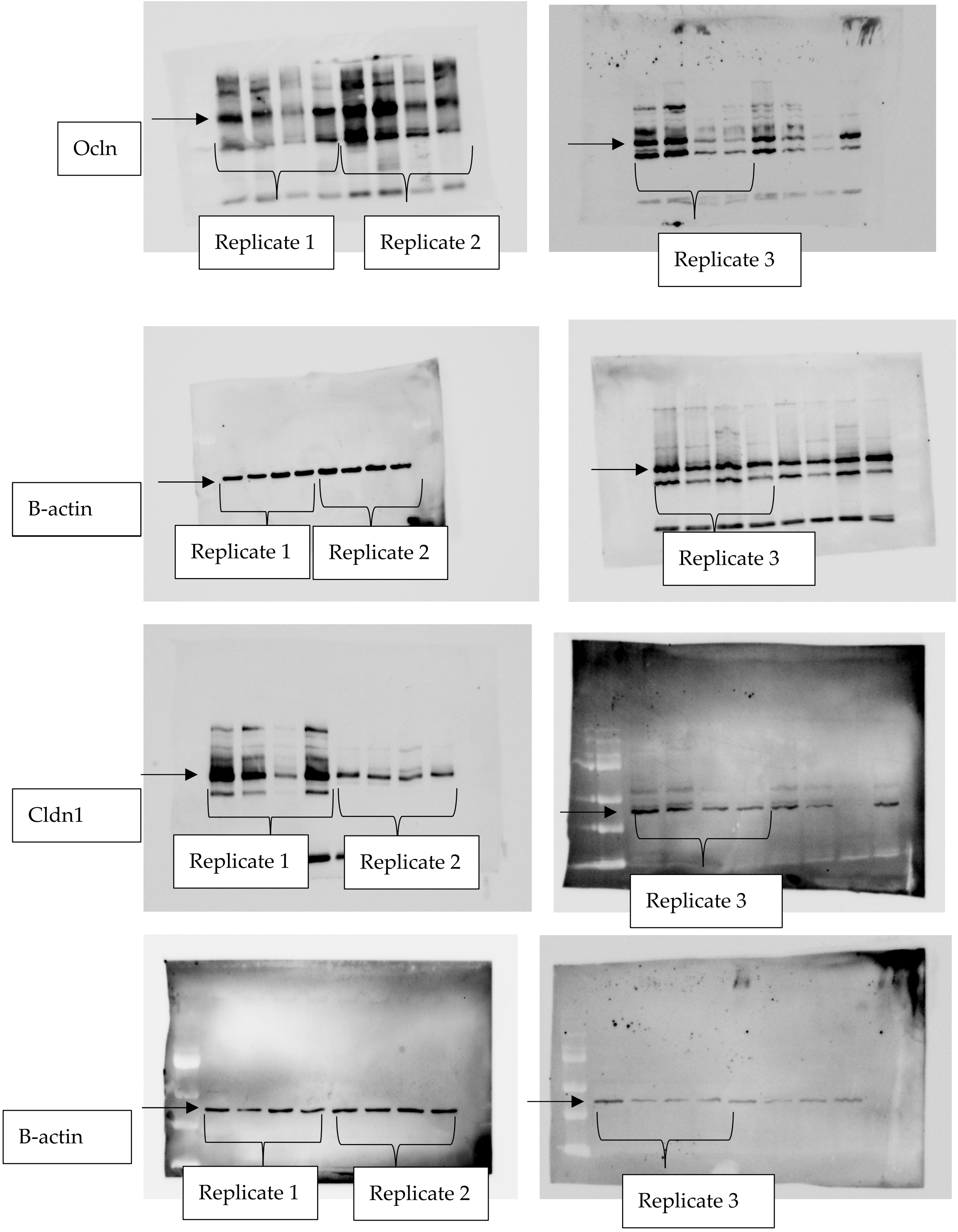

Supplement: Supplementary file 1 [file biology-13-00955-s001.zip › biology-3285827-supplementary.pdf]
